# PD51 Effectiveness And Safety Of Vitamin D For COVID-19: A Living Evidence Synthesis Informing A Health Technology Assessment Report

**DOI:** 10.1017/S0266462324003106

**Published:** 2025-01-07

**Authors:** Juan Antonio Blasco-Amaro, Trinidad Sabalete-Moya, Rocío Rodríguez-López, Maria X. Rojas

## Abstract

**Introduction:**

An evidence synthesis developed to inform decision-making on the use of vitamin D for preventing and treating COVID-19 showed that current available evidence is of low to very low quality. We set up a rigorous living evidence to inform health decisions (LE-IHD) approach to provide timely updates of this health technology assessment (HTA) report and aid decision-making.

**Methods:**

Following the LE-IHD framework, we developed a baseline synthesis and evidence monitoring on the effects of high-dose vitamin D for the prevention and treatment of severe COVID-19 on all-cause mortality, COVID-19-related hospitalization, intensive care unit admission, length of hospital stay, quality of life, adverse events, and long COVID-19. The evidence identification, screening, and selection processes were supported by Epistemonikos technological enablers and the Living Overview of Evidence platform. We searched for ongoing studies in trial registries every three months. New eligible studies were assessed using a systematic and reproducible process to update the HTA report.

**Results:**

For the baseline synthesis we identified nine randomized control trials (RCTs) assessing high dose vitamin D2, vitamin D3, and their metabolites, which provided very low quality evidence on all the outcomes of interest. Up to date evidence monitoring identified seven studies reporting on all-cause mortality and intensive care unit admission, eight studies reporting on length of hospital stay, and six studies reporting on adverse events. The living evidence synthesis has been updated twice. At the time of the conference, we will report on 10 months of monitoring results and any substantial updates to the HTA report.

**Conclusions:**

For HTA reports based on low and very low quality evidence (uncertain results), the living evidence approach allows for timely updating of conclusions. The LE-IHD framework facilitates the planning and execution of living evidence syntheses to inform health decisions. This living evidence synthesis is being developed as part of a project to strengthen decision-making capacity in the Spanish health system.

